# Modeling the Energy Performance of LoRaWAN

**DOI:** 10.3390/s17102364

**Published:** 2017-10-16

**Authors:** Lluís Casals, Bernat Mir, Rafael Vidal, Carles Gomez

**Affiliations:** Department of Network Engineering, Universitat Politècnica de Catalunya/Fundació i2Cat, C/Esteve Terradas, 7, 08860 Castelldefels, Spain; lluis.casals@entel.upc.edu (L.C.); bernatmir13@gmail.com (B.M.); rafael.vidal@entel.upc.edu (R.V.)

**Keywords:** LoRaWAN, LoRa, energy, energy modeling, performance evaluation, Internet of Things, IoT, smart cities, LPWAN

## Abstract

LoRaWAN is a flagship Low-Power Wide Area Network (LPWAN) technology that has highly attracted much attention from the community in recent years. Many LoRaWAN end-devices, such as sensors or actuators, are expected not to be powered by the electricity grid; therefore, it is crucial to investigate the energy consumption of LoRaWAN. However, published works have only focused on this topic to a limited extent. In this paper, we present analytical models that allow the characterization of LoRaWAN end-device current consumption, lifetime and energy cost of data delivery. The models, which have been derived based on measurements on a currently prevalent LoRaWAN hardware platform, allow us to quantify the impact of relevant physical and Medium Access Control (MAC) layer LoRaWAN parameters and mechanisms, as well as Bit Error Rate (BER) and collisions, on energy performance. Among others, evaluation results show that an appropriately configured LoRaWAN end-device platform powered by a battery of 2400 mAh can achieve a 1-year lifetime while sending one message every 5 min, and an asymptotic theoretical lifetime of 6 years for infrequent communication.

## 1. Introduction

Low-Power Wide Area Networks (LPWAN) define a category of wireless communication technologies that have recently gained significant momentum. Industry, academia and standards development organizations have devoted significant efforts to LPWAN in the last years [1,2]. Such technologies typically offer a link range of one or more kilometers, while a single infrastructure element (often called a gateway) is capable of supporting hundreds of thousands of devices such as sensors and actuators [3,4]. Therefore, LPWAN technologies enable Internet of Things (IoT) applications, such as smart cities, by means of low cost and low complexity infrastructure.

Among LPWAN technologies, LoRaWAN (and its related modulation, LoRa), is perhaps the technology that has attracted the attention of most academic work as of the writing of this paper [5,6,7,8,9,10,11,12,13,14,15,16,17,18,19,20,21,22,23,24,25,26]. This can be explained by the public availability of its specifications [27], the availability of certified hardware [28] and the fact that LoRaWAN communication can be enabled without the need to establish a relationship with an operator. 

Many LoRaWAN devices, such as sensors or actuators, will typically be battery-operated. Therefore, it is crucial to investigate the characteristics of LoRaWAN energy consumption. However, published works only focus on this topic to a limited extent, providing only rough estimates on parameters related with LoRaWAN energy performance, while not considering realistic behavior of LoRaWAN device hardware, as well as the impact of the main LoRaWAN parameters and mechanism settings [13,22,25,26].

In this paper, we present analytical models that characterize device current consumption, device lifetime and energy cost of data delivery with LoRaWAN. For a realistic analysis, the models are derived based on measurements performed on a currently prevalent LoRaWAN platform. The models developed allow studying the impact of relevant LoRaWAN parameters and mechanisms such as data rates, acknowledged transmission and payload size, as well as Bit Error Rate (BER) and collisions, on LoRaWAN energy performance. Evaluation results illustrate energy performance and trade-offs of LoRaWAN. Among others, we have found that using an appropriate configuration a LoRaWAN end-device platform powered by a 2400 mAh battery can achieve a 1-year lifetime while sending one message every 5 min, and an asymptotic theoretical lifetime of 6 years as communication becomes infrequent. 

The remainder of the paper is organized as follows. Section 2 reviews related work. Section 3 overviews LoRaWAN, describing its general architecture and focusing on its physical and MAC layers. Section 4 models LoRaWAN end-device current consumption, end-device lifetime, and energy cost of data delivery, whereas Section 5 provides evaluation results obtained by using the models presented. Section 6 concludes the paper.

## 2. Related Work

Most of the research done on LoRa/LoRaWAN has focused on features such as coverage, robustness, capacity, scalability, delay and throughput [3,5,6,7,8,9,10,11,12]. However, a characteristic such as energy consumption, which is crucial considering that many LoRa/LoRaWAN devices will not be grid-powered, has received limited attention. We next review the literature on LoRaWAN energy consumption. We first focus on current consumption details of LoRa/LoRaWAN devices reported in published works, and secondly we discuss the few existing models of LoRa/LoRaWAN energy consumption, node lifetime or energy cost of data delivery. 

Several works provide current consumption data of LoRa/LoRaWAN devices, obtained from a datasheet or by empirical means [12,13,14,15,16,17,18,19,20,21,22]. Such details, which are summarized in Table 1, correspond to sleep, transmission and reception device states. As it can be seen, sleep current ranges from 7.66 μA to 34 mA (or between 30.9 μA and 3.4 mA excluding LoRa-only and custom devices). Sleep current for the considered hardware platforms is up to several orders of magnitude greater than that of their transceivers (see Table 2), which can be near or even lower than 1 μA. One important conclusion is that current LoRa/LoRaWAN nodes are far from the degree of optimization exhibited by platforms that use other low power technologies. For example, IEEE 802.15.4 and Bluetooth Low Energy (BLE) commercial devices feature a sleep current near 1 μA [23,24]. Therefore, in order to achieve attractive node lifetime figures (e.g., in the order of years), current LoRaWAN nodes need batteries with greater capacity than typical button cell batteries, e.g., of AA type, which however have bigger size and are more expensive. We attribute the sleep current in LoRaWAN devices to suboptimal hardware integration of device components, e.g., the microcontroller and the transceiver.

Based on the characterization of sleep, transmit and receive states of a LoRa/LoRaWAN device, a few analytical models of LoRa/LoRaWAN energy consumption, node lifetime or energy cost of data delivery have been published [13,22,25,26]. However, these models are too simple, since there exist several other states for a LoRa/LoRaWAN device involved in a communication that need to be considered (see Section 4). Next, we briefly present the main results and other limitations of these works. An accurate calculation of message transmission time is only provided in [25], however the study only focuses on LoRa, and therefore it does not model the MAC layer mechanisms defined in LoRaWAN, such as use of acknowledgments, receive windows, and retransmissions (see Section 3). In [13], which focuses only on optimizing downlink communications, fixed LoRaWAN settings (i.e., a single DR and acknowledged transmission) are considered. Energy consumption of 0.05–0.44 mJ and a battery lifetime between 13 and 1 year, respectively, are obtained for a device running on two AA batteries, when transferring data from 1 to 10 times per hour. However, one of the most important values used in the model, the sleep current (of 2 µA), is without explanation significantly lower than the corresponding value in the datasheet published by the manufacturer (i.e., 30.9 µA). In [26], only the energy related to the activation of a LoRaWAN node by using the On-The-Air Activation (OTAA) mode is modeled. In [22], the battery capacity consumed during a day by a device is calculated assuming 100 events detected and 10 frame transmissions performed by the device. The result is 222.66 µAh, which corresponds to a lifetime of 21.5 months for a 150 mAh button cell battery. Neither the impact of using acknowledgments, nor the influence of the DR configured are considered. Finally, the impact of errors due to corruption on LoRaWAN energy performance has not been modeled in publicly available works. 

Based on the literature review, we conclude that, to the best of our knowledge, this paper is the first one that provides a detailed analytical model of node energy consumption, node lifetime and energy cost of data delivery, considering real LoRaWAN device hardware behavior and physical and MAC layer parameters and mechanisms. 

## 3. LoRaWAN Overview

In this section, we present fundamental LoRaWAN characteristics. We describe the protocol architecture as well as the physical and MAC layers, highlighting the mechanisms, procedures and key parameters that are relevant in the scope of this paper. This section is organized in three subsections. The first one provides a general LoRaWAN overview, whereas the remaining two subsections focus on physical and link layer functionality. 

### 3.1. LoRaWAN General Overview

LoRaWAN is a wireless communication technology designed to achieve long range while consuming low power, based on a one-hop radio system. This approach allows overcoming some important issues related with deployment complexity and low energy routing protocols, among others [18]. On the other hand, LoRaWAN exhibits communication constraints, which may limit the suitability of this technology for present and future IoT applications. 

**Figure 1:**
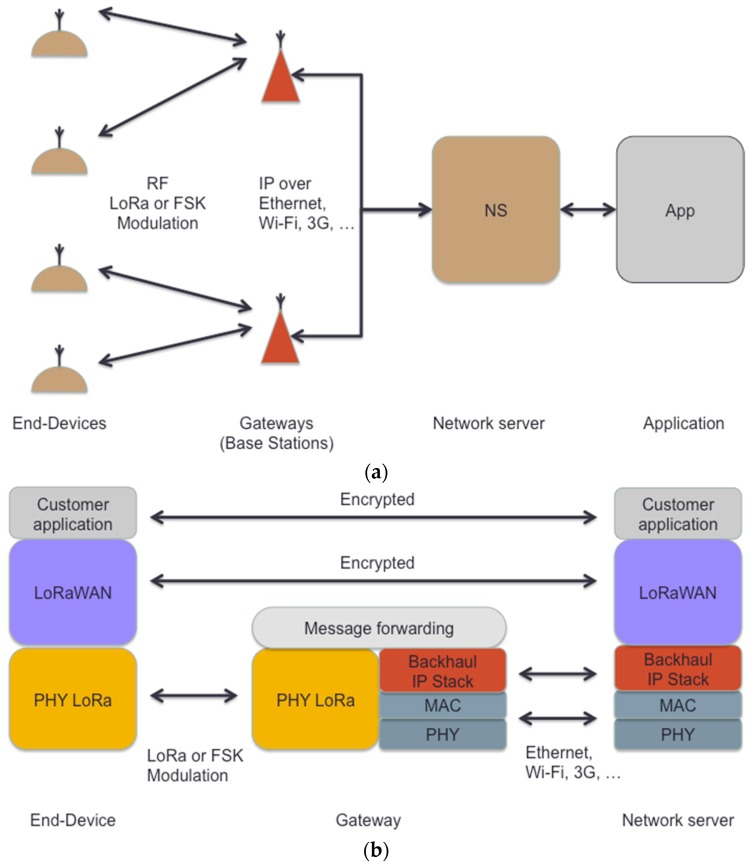
LoRaWAN (**a**) system and (**b**) protocol architecture.

A LoRaWAN network is based on a star-of-stars topology composed of three basic elements: end-devices, gateways and a central network server (see Figure 1a) [27]. End-devices, which may correspond to e.g., sensors or actuators, communicate with the network server through one or more gateways, while the network server sends messages to end-devices through a specific gateway. End-devices use the LoRa physical layer to exchange messages with the gateway, while the gateway and the network server communicate over an IP-based protocol stack (Figure 1b).

LoRAWAN provides end-to-end encryption and data integrity. Upper layer payloads are encrypted by means of an Application Session Key, which provides confidentiality. On the other hand, a Network Session Key is used to provide data integrity over the ciphertext payload and a subset of header fields [27].

LoRaWAN specification defines three functionality classes: Class A, Class B and Class C. Class A must be implemented by all LoRaWAN devices, and is also known as basic LoRaWAN. Class A allows bidirectional communication between an end-device and the network server, which is scheduled by the end-device based on its needs. In this functionality class, downlink transmission (i.e., from the network server to the end-device) can only occur after an uplink transmission (i.e., from the end-device to the network server).

Class B is based on class A; however, it supports additional downlink transmission opportunities at prescheduled times. On the other hand, Class C allows downlink transmission at any time, except when the end-device is transmitting. Class C end-devices consume greater power to operate, but offer the lowest latency, compared with Class A and Class B end-devices. Both Class B and Class C are optional, and are in consequence not typically supported by LoRaWAN devices.

The study presented in this paper focuses on Class A LoRaWAN operation. The next two subsections present physical layer and MAC layer details for this LoRaWAN mode.

### 3.2. LoRaWAN Physical Layer

#### 3.2.1. Physical Layer Main Features

LoRa modulation and Gaussian Frequency Shift Keying (GFSK) provide the physical transmission support for communication between the end-device and the gateway. LoRa modulation is based on chirp spread spectrum mechanism [38]. Each LoRa symbol is composed of 2^SF^ chirps, where SF represents the corresponding spreading factor [7]. The use of six orthogonal SFs in the range of 7 to 12, which provide different Data Rates (DRs), results in a better spectral efficiency and an increased network capacity. LoRa modems also use forward error correction, adding a small overhead to the transmitted message, which provides recovery features against bit corruption. This is implemented through different Coding Rates (CRs), from 4/5 to 4/8 (denoted CR = 1 to CR = 4, respectively). On the other hand, to avoid issues regarding drift of the crystal reference oscillator, a low data rate optimization mechanism is applied, which adds a small overhead to increase robustness to frequency variation over the timescale of the LoRa message [38]. This is done for SF = 11 and SF = 12.

The Radio Frequency (RF) band to be used depends on the country where LoRaWAN is deployed. Our study considers operation in the European region, in the EU863–870 Industrial Scientific Medical (ISM) band, where three default channels are defined: 868.10, 868.30, 868.50 MHz. Each one of these channels has a bandwidth of 125 kHz, uses the LoRa modulation, and must allow data rates from 0.3 kbps to 5 kbps (DR0 to DR5, see Table 3). These channels must be implemented in every end-device within the European region. LoRaWAN also enforces duty-cycle limitations stated by ETSI regulations. For the sub-band comprising between 868.0 MHz to 868.6 MHz, the duty-cycle must be <1%. Then, this duty cycle has to be distributed in the three channels of this sub-band [18]. Note, however, that ETSI regulations limit the duty-cycle over one-hour intervals, whereas LoRaWAN enforces compliance with such limitation over the interval between transmission of a message and the next one. The reference indices for each data rate (DR), and the corresponding spreading factor (SF), bandwidth and modulation configuration, and resulting physical bit rate are shown in Table 3, which also includes the details for optional DR6 and DR7.

**Figure 2:**
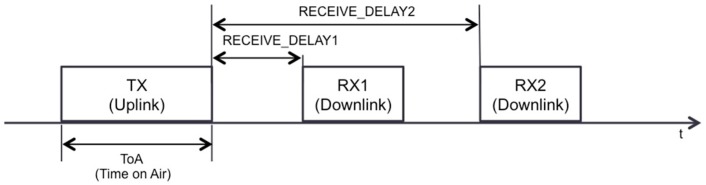
Basic transmission scheduling in LoRaWAN Class A functionality.

As mentioned earlier, Class A LoRaWAN functionality focuses on the end-device, which has the main role in message exchanges: all transactions are started by the end-device, whereas the network server can only transmit in one of two downlink slots, called receive windows (RX1 and RX2, in Figure 2), opened by the end-device, following a previous uplink transmission. Therefore, any message the network server has to transmit after RX2 must wait until the next receive window to be sent.

An end-device uses a different frequency channel for each message transmission, by following a pseudo-random channel sequence. This procedure makes communication robust against interference or other radio propagation impairments (e.g., multipath, etc.). 

#### 3.2.2. Physical Layer Message Format

**Figure 3:**
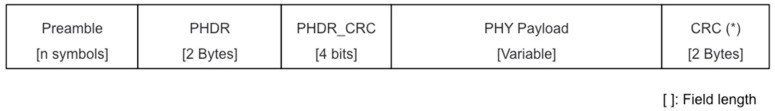
LoRaWAN physical layer message format. (*) Cyclic Redundancy Check (CRC) field is only present in uplink transmissions. The preamble length is configurable; for the device used in this study, preamble length is *n* = 8 symbols.

LoRaWAN specification defines a physical layer message that comprises a preamble, a physical header (PHDR), a physical header Cyclic Redundancy Check (PHDR_CRC), a physical payload (PHY Payload), and an error detection tail (CRC) [27]. Figure 3 shows the physical layer message structure. PHDR and PHDR_CRC fields have a combined total size of 20 bits. Additionally, 2 bytes of CRC are present only in uplink messages, thus the downlink is optimized for low transmission time.

#### 3.2.3. Receive Window Parameters

The DR, the frequency channel and the time between the end of an uplink transmission and the start time for the first and second receive windows (*RECEIVE_DELAY1* and *RECEIVE_DELAY2*, respectively, see Figure 2) are defined in the LoRaWAN regional parameters document [39]. Table 4 shows the main basic physical layer parameters, and their default values.

The DR to be used in the first receive window (RX1) can be set as the uplink DR minus the DR offset RX1 parameter, *RX1DROffset*, if the latter is equal to or less than the DR of the uplink; otherwise, the DR for the first receive window will be DR0. *RX1DROffset* can take values in the range of 0 to 5. Since *RX1DROffset* has a default value of zero, the DR for the first receive window is by default the same one used in the last uplink transmission. 

The frequency channel used in the first receive window is the same as the one used for the preceding uplink transmission, while the second receive window uses a fixed frequency and data rate configuration (by default, frequency 869.525 MHz, and DR0). These parameters can be customized in the hardware platform used in the study. However, other platforms (e.g., RN2483, [36,37]) do not support such configurability.

The duration of a receive window has to be at least the time required to effectively detect a downlink preamble [27]. If a preamble is detected during a receive window, the end-device radio remains active until the reception of a complete message. If this is done successfully in the first window, the end-device does not use the second receive window. If a transmission is not detected within a receive window, the radio transceiver and, possibly, the microcomputer chip that support the device will be in an active state, with a significant current consumption, for a certain period of time during the window. This time is an important factor for the end-device lifetime and it is strongly related to the specific transceiver hardware used (see Section 4).

In the hardware platform we have used in this paper, the time spent in preamble detection in a first receive window is determined as the symbol timeout parameter (*SymbTimeout*), which is set to 8 symbols for DR0 (SF12) and DR1 (SF11), and 12 symbols for the rest of DRs. On the other hand, for the second receive window, a feature called Channel Activity Detection (CAD) is used. This mechanism reduces channel listening time. If no incoming signal is detected, the end-device decides earlier to stop preamble detection, thus saving radio on time and current consumption.

### 3.3. LoRaWAN MAC Layer

#### 3.3.1. MAC Message Format

**Figure 4:**
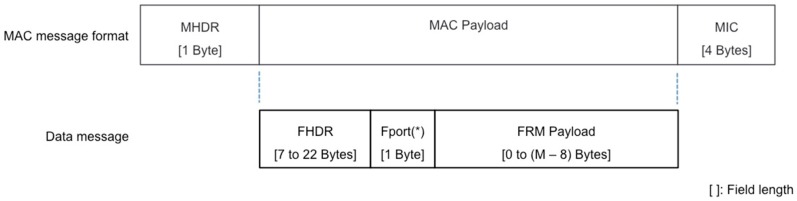
LoRaWAN Medium Access Control (MAC) message format. The (*) FPort field is present when the frame payload (FRM Payload) field contains data (i.e., it has a non-zero length). *M* denotes the maximum size of the MAC Payload field. The frame header (FHDR) field has a size of 7 bytes if it does not contain options, and up to 22 bytes when options are used (with an option size of up to 15 bytes). In this paper, we assume that options are not present in data messages.

LoRaWAN defines a set of MAC message types that are transmitted as payload of a physical layer message (i.e., are carried in the PHY Payload field). Three basic types of MAC messages are defined: (i) the Join message; (ii) the Confirmed Data message and (iii) the Unconfirmed Data message. The format for a MAC message is shown in Figure 4. The MAC message comprises: (i) the MAC Header (MHDR), which indicates the type of MAC message; (ii) the MAC payload, which can carry application data or a Join message; and (iii) the Message Integrity Code (MIC), which allows a receiver to check the integrity of a MAC message received. On the other hand, data messages can carry MAC commands in the frame payload (FRM Payload), which can also be carried in the frame header (FHDR), depending on the FPort value. MAC commands are intended to configure radio and MAC layer parameters. An important subfield in the FHDR is a bit that allows acknowledging the last Confirmed Data message received. 

#### 3.3.2. Transmission and Retransmission Procedure

When an end-device transmits an uplink Confirmed Data message, it expects to receive a downlink acknowledgment message in one of the next two receive windows. If the acknowledgment is not received, the end-device retransmits the same message until an acknowledgment is received or until a maximum number of MAC layer transmission attempts for the message (the recommended default number being 8) is reached. Each transmission attempt is done in a different channel, which is randomly selected from the channels available in the subband used. The DR to be used is recommended to follow the next rules. The 1st and 2nd transmission attempts of a confirmed message are done by using the same DR, the 3rd and 4th attempts use the next lower data rate (or DR0 if it was the DR previously used), and so on, until the 8th transmission attempt. After 8 transmission attempts of the same confirmed message without an acknowledgment, the MAC layer should return an error code to the upper layer (i.e., the application layer). Each retransmission is started after an acknowledgment timeout (*ACK_TIMEOUT*) period, which is initiated at the start time of the last 2nd receive window, and is defined as a random delay between 1 and 3 seconds, by default [39]. 

## 4. Modeling LoRaWAN End-Device Current Consumption

In this section, we present models of crucial LoRaWAN energy performance parameters such as end-device current consumption, end device lifetime, and energy efficiency of data delivery. We assume a class A end-device that periodically transmits an uplink data message (e.g., a notification that carries a sensor reading). In the models, we consider the impact of bit errors. For the sake of tractability and clarity, we assume a uniform BER that refers to the residual BER after application of physical layer error correcting techniques, equivalent to the residual BER that corresponds to message loss rate due to non-ideal link quality. The section is divided in two subsections, which offer the aforementioned models for unacknowledged and acknowledged transmission, i.e., the transmission of unconfirmed and confirmed data messages, respectively. We develop the models for all DRs that are mandatory (i.e., from DR0 to DR5), as well as for DR6. 

### 4.1. Unacknowledged Transmission 

**Figure 5:**
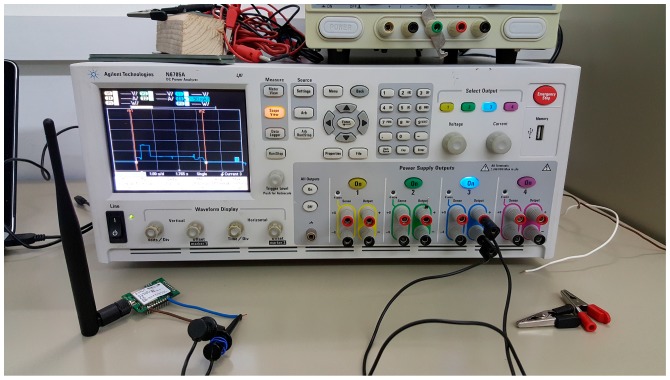
Experimental setup for current measurements of the MultiConnect mDot LoRaWAN end-device module (on the left) using an Agilent N6705A power analyzer.

Our first goal is modeling the average current consumption of an end-device in the unacknowledged approach, denoted *I_avg_unACK_*. In order to determine this parameter, we first derive a profile of the different states traversed by the end-device, as well as the duration and the current consumed in each state. In order to realistically model the end-device behavior, and without loss of generality, we develop the model based on measurements from a real LoRaWAN testbed. The measurement setup is shown in Figure 5. We use the MultiConnect mDot platform from Multitech [29] as our reference end-device platform for the model, since it is a popular platform, and it is based on the also widely used SX1272 transceiver [33] (see Table 1). While other LoRaWAN end-device platforms might exhibit differences with the mDot platform (e.g., due to their internal architecture), we understand that our model captures the main states of a LoRaWAN end-device. On the other hand, it must be noted that the mDot platform offers low current consumption decrease (of ~3%, and only in the transmit state) when the voltage applied is reduced from 5 V to 3.3 V, the latter being the lowest voltage that allows the device to operate [29]. However, other platforms may not offer the same current consumption stability as battery voltage decreases over time. 

In the measurements, the transmit power of the end-device is set to 11 dBm, which is the default value for this parameter. The gateway is a Kerlink LoRa IoT Station platform [40]. Both the end-device and the gateway are located in an indoor scenario, where the distance between the end-device and the gateway is 2 m. In the measurements, data messages carry a frame payload of the maximum size allowed for each DR in the EU band. 

We assume a periodic behavior for the LoRaWAN end-device, therefore we model its current consumption during one period. Each period comprises a data message transmission by the end-device (including the related procedures required to enable such transmission), otherwise the device is in sleep mode. Note that, for an end-device in unacknowledged transmission, current consumption is independent of the BER; that is, regardless of whether channel errors take place in the communication, the end-device will consume the same amount of energy for any transmission, since there will not be retransmissions in unacknowledged transmission. Time and current consumption measurement results provided in this section are obtained from several measurements for each tested configuration within a notification period. We found negligible differences within each set of measurements for each configuration. 

**Figure 6:**
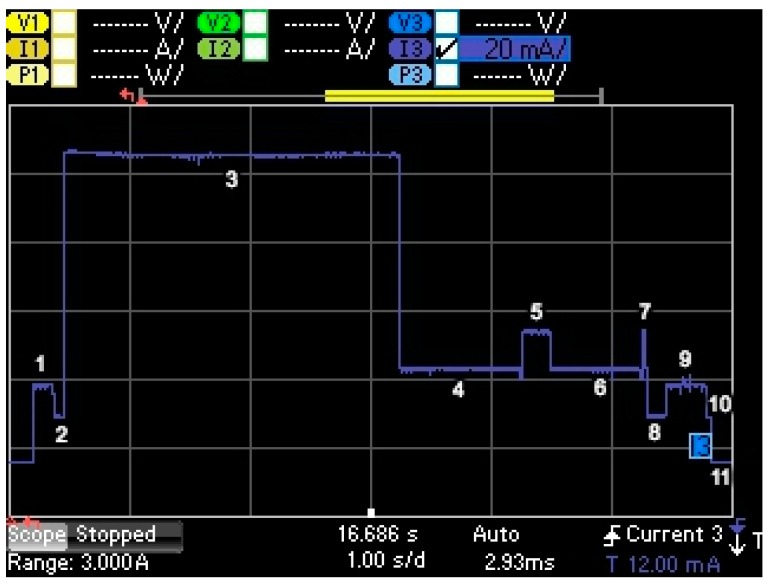
Current consumption profile of a MultiConnect mDot LoRaWAN end-device performing an unacknowledged transmission with DR0. The data message transmitted has a FRM Payload size of 51 bytes (i.e., maximum possible size for DR0).

Figure 6 illustrates the current consumption profile of an unacknowledged transmission performed by the MultiConnect mDot LoRaWAN end-device configured to use DR0 (note that the states traversed and behavior observed are the same for all DRs, except for the duration of some intervals). Table 5 defines and describes the different states involved in an unacknowledged transmission, along with the variables that represent the duration and current consumption of each state. Initially, the end-device is in sleep mode, which is characterized by a current consumption three orders of magnitude below that of the rest of states. When the end-device starts the procedure to perform the transmission, it first wakes up (state 1), next the radio interface is prepared for activity (state 2), and then the end-device transmits the data unit via the radio interface (state 3). After the transmission, the end-device disables radio activity and waits (state 4) until it sets the radio into receive mode and remains in the same state for the duration of the first receive window (state 5). Since no incoming preamble is detected, the first receive window is closed, and the end-device waits (state 6) until the start of the second receive window. During the latter, the end-device radio is turned on for possible incoming data units, until the second receive window is closed due to absence of incoming data (state 7). Note that the shorter duration of the second receive window is due to use of the CAD mechanism (see Section 3.2.3), whereby the end-device stops preamble detection much earlier than in the first receive window if no incoming signal is detected. After that, the radio interface is turned off (state 8), a postprocessing interval follows (state 9), and the end-device executes a turn off sequence (state 10), prior to returning to the sleep state (state 11). One additional consideration is that we have not identified any specific state due to the internal communication, which takes place via Serial Peripheral Interface (SPI), between the main microcontroller and the radio interface of the mDot platform. This is consistent with the submillisecond latency of payload transmission from the microcontroller to the radio interface, for the range of payload sizes in LoRaWAN, that is typical of SPI.

Let *T_Notif_* be the time between two consecutive periodic message transmissions performed by the end-device, i.e., the notification period. Let *T_i_* and *I_i_* denote the duration and current consumption of state *i* in Table 5. *I_avg_unACK_* can thus be calculated as shown in Equation (1):

(1) $$I_{avg\_unACK}=\;\frac{1}{T_{Notif}}\overset{N_{states}}{\underset{i=1}{\sum }}T_{i}\cdot I_{i}$$

where *N_states_* is 11 in unacknowledged transmission. Note that *T_sleep_* can be obtained as:

(2) $$T_{sleep}=T_{Notif}-T_{act}$$

where *T_act_* denotes the sum of the durations of all states related with transmission activities, i.e., all states except the sleep interval:

(3) $$T_{act}=T_{wu}+T_{pre}+T_{tx}+T_{w1w}+T_{rx1w}+T_{w2w}+T_{rx2w}+T_{off}+T_{post}+T_{seq}$$

The durations *T_tx_*, *T_rx_*_1*w*_, and *T_w_*_2*w*_ are variable and depend on the DR in use. *T_w_*_2*w*_ actually depends on *T_rx_*_1*w*_, and can be obtained as:

(4) $$T_{w2w}=RECEIVE\_DELAY\_2-RECEIVE\_DELAY\_1-T_{rx1w}$$

Although *T_rx_*_2*w*_ also depends on the DR, the end-device platform in our experiments uses a fixed setting for the second receive window (which corresponds to DR0), and thus the measured value for *T_rx_*_2*w*_ is also constant. We next provide the models to derive *T_tx_*, *T_rx_*_1*w*_ and *T_rx_*_2*w*_.

In order to determine the time needed to transmit a data message via the radio interface, *T_tx_*, we take into acount LoRaWAN procedures, LoRa modulation details and the corresponding regional parameters. *T_tx_* can be expressed in terms of the time required to transmit both the preamble and the physical message, denoted *T_preamble_* and *T_PHYMessage_*, respectively, as follows [38]:

(5) $$T_{tx\;}=\;T_{preamble\;}+T_{PHYMessage}$$

*T_preamble_* can be obtained as shown next [33]:

(6) $$T_{preamble}=T_{sym}\cdot \left(N_{pre}+4.25\right)$$

where *N_pre_* is the programmed number of symbols to be used by the radio transceiver, the actual physical length of the preamble is (*N_pre_* + 4.25) [7], and *T_sym_* is the time of a symbol (in seconds), which depends on the SF and the channel bandwidth (BW, in Hz), as follows [38]:

(7) $$T_{sym}=\frac{2^{SF}}{BW}$$

On the other hand, $T_{PHYMessage}$ (in seconds) can be evaluated similarly:

(8) $$T_{PHYMessage}=T_{sym}*N_{PHY}$$

where $N_{PHY}$ indicates the number of symbols transmitted as the physical message (excluding the preamble), and it can be determined as follows [38]:

(9) $$N_{PHY}=8+\max\left[ceil\left[\frac{28+8\cdot PL+16\cdot CRC-4\cdot SF}{4\cdot \left(SF-2\cdot DE\right)}\right]\cdot \left(CR+4\right),0\right]$$

In (9), *SF* corresponds to the spreading factor and can take values from 7 to 12 (which correspond to data rates from DR5 to DR0, respectively); *CR* denotes the coding rate and can take values from 1 to 4, for 4/5 to 4/8 coding rate, respectively; *PL* indicates the physical payload length, in bytes. *CRC* indicates the presence or not of the CRC field in the physical message (*CRC* is set to 0 if the CRC field is not present; otherwise, *CRC* is equal to 1); finally, *DE*, which indicates whether the mechanism to avoid issues regarding drift of the crystal reference oscillator is used or not, takes value 1 for SF12 and SF11 (i.e., it is used for the lowest data rates), and value 0 for the rest of SFs. Equations (5)–(9) can be used to model the duration of both uplink and downlink transmissions (e.g., the latter may correspond to acknowledgments sent in response to uplink data messages, see Table 6).

After modeling *T_tx_*, we proceed to determine the duration of a receive window when no preamble is detected, which occurs in both receive windows in unacknowledged transmission. We next model the behavior of the hardware module used in our experiments in each receive window. 

For the first receive window, *T_rx_*_1*w*_ can be determined as follows:

(10) $$T_{rx1w}=N_{dsym}\cdot T_{sym}$$

The end-device stays in receive mode for the duration of $N_{dsym}$ symbols $N_{dsym}$ is 8 symbols for SF = 12 and SF = 11, and 12 symbols for the rest of SFs.

For the second receive window, the receiver is active during a fraction of a CAD state (see Section 3.2.3). This fraction has a duration, denoted *T_rx_*_2*w*_, that can be calculated as shown next:

(11) $$T_{rx2w}=\frac{2^{SF}+32}{BW}$$

After providing the models for determining *T_tx_*, *T_rx_*_1*w*_ and *T_rx_*_2*w*_, Table 6 summarizes their main values, along with relevant parameter settings used in our end-device platform. For *T_rx_*_2*w*_, we include the corresponding values for the different DR settings possible. However, in our experiments, only the *T_rx_*_2*w*_ value corresponding to DR0 was used. 

Once all variables required to compute *I_avg_unACK_* are determined, we can calculate the theoretical lifetime of a battery-operated end-device that performs unacknowledged transmissions, denoted *T_lifetime_unACK_*, can be obtained on the basis of the battery capacity, *C_battery_* (expressed in mA·h), as shown next:

(12) $$T_{lifetime\_unACK}=\;\frac{C_{battery}}{I_{avg\_unACK}}$$

Note that the above theoretical end-device lifetime calculation assumes an ideal battery with a linear behavior, whereas the characteristics of a real battery degrade over time. Therefore, the calculated end-device lifetime results provided in this paper provide an upper bound on the actual end-device lifetime that can be expected.

Finally, another important performance parameter is the energy cost of data delivery, *EC_delivery_unACK_*, which provides the energy consumed by the end-device per each delivered bit of data payload in unacknowledged mode, as shown below:

(13) $$EC_{delivery\_unACK}=\;\frac{I_{avg\_unACK}\cdot V\cdot T_{notif}}{E\left[l_{delivery\_unACK}\right]}$$

where *V* denotes the voltage and *E*[*l_delivery_unACK_*] indicates the expected amount of data successfully delivered by the end-device per data frame transmitted. Note that in the previous equation, the numerator computes the energy consumed by the device during *T_notif_*. 

Let *l_pay_* be the FRM Payload field size (i.e., the amount of data carried in the payload of the data message sent by the end-device), and let *l_Data_* be the total size of the data message, including all headers. Let *b* denote the BER as introduced in the first paragraph of Section 4. Since in unacknowledged transmission there is a single transmission attempt, which may suffer bit errrors, *E*[*l_delivery_unACK_*] is determined as:

(14) $$E\left[l_{delivery\_unACK}\right]=\;l_{Pay}\cdot {\left(1-b\right)}^{l_{Data}}$$

Finally, let us assume that collisions may occur, i.e., an end-device message transmission may overlap with messages transmitted by other end-devices connected to the same gateway. Let *p_coll_* be the probability that a data message transmitted by an end-device collides with at least another message transmission. In order to capture impact of collisions on *E*[*l_delivery_unACK_*], Equation (14) can be extended as follows:

(15) $$E\left[l_{delivery\_unACK}\right]=\;l_{Pay}\cdot {\left(1-b\right)}^{l_{Data}}\cdot \left(1-p_{coll}\right)$$

Note that, as already introduced, *b* corresponds to the residual BER after application of physical layer error correcting techniques, equivalent to the residual BER that corresponds to message loss rate due to non-ideal link quality. In this paper, we assume that CR = 4/5, since it is the default CR in LoRaWAN, except for the 20-bit physical header, where CR = 4/8 is used. For CR = 4/5, a parity bit is added to each group of 4 bits from the physical layer message to be transmitted. Assuming that an error-correcting code is used for CR = 4/8 (e.g., a Hamming code [41]) and the range of BER values for reasonably useful links, and given the short size of the physical header, we approximate the relationship between *b* and the physical layer BER, denoted *b_phy_*, as shown in the next two equations. Let *p_loss_* be the probability that a transmitted message is affected by at least one bit error, and therefore the message is lost, and let *l_phy_header_* be the 20-bit header size. Therefore:

(16) $$p_{loss}=\;1-{\left(1-b\right)}^{l_{Data}}=1-{\left(1-b_{phy}\right)}^{\frac{5}{4}\cdot \left(l_{Data}-l_{phy\_header}\right)}$$

(17) $${\left(1-b\right)}^{l_{Data}}={\left(1-b_{phy}\right)}^{\frac{5}{4}\cdot \left(l_{Data}-l_{phy\_header}\right)}$$

### 4.2. Acknowledged Transmission 

We next model end-device average current consumption in the acknowledged transmission approach, based on the corresponding current consumption profile of the same hardware platform as in the previous subsection. 

In this approach, the end-device may behave in two different ways, since the acknowledgment may be transmitted in the first receive window or in the second one. In our model, we consider both options. We initially assume BER = 0, and we subsequently extend the model in order to consider a non-zero BER. Therefore, the average current consumption of an end-device in acknowledged mode, denoted *I_avg_ACK_*, can be obtained as shown in the next equation:

(18) $$I_{avg\_ACK}=\;p_{1win}\cdot I_{avg\_ACK\_1}+p_{2win}\cdot I_{avg\_ACK\_2}$$

where *I_avg_ACK__*_1_ and *I_avg_ACK__*_2_ denote the average current consumption of the end-device when the acknowledgment is received in the first and in the second receive window, respectively, and *p*_1*win*_ and *p*_2*win*_ represent their corresponding probabilities. The LoRaWAN specification offers freedom for network managers and implementers to apply the policy that best suits the requirements of a specific deployment. Therefore, since there is no specific priority by default for the two receive windows, we assume that an acknowledgment may be received by an end-device in the first or in the second receive window with the same probability (i.e., *p*_1*win*_ = 0.5 and *p*_2*win*_ = 0.5).

**Figure 7:**
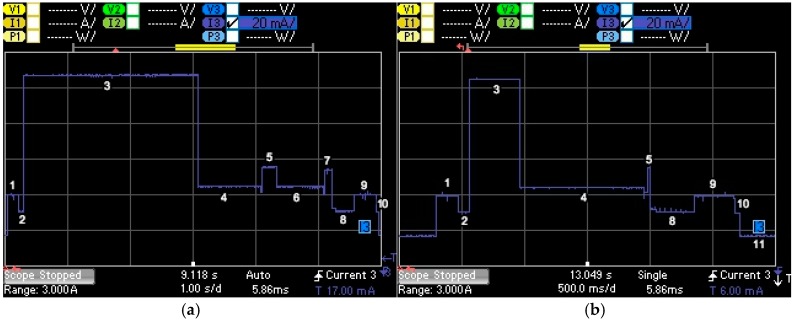
Current consumption profile of a MultiConnect mDot LoRaWAN end-device performing an acknowledged transmission: (**a**) with DR0 (left), (**b**) with DR5 (right). In the former, the acknowledgment is received by the end-device in the second window, whereas in the latter the acknowledgment is received in the first window. The data message transmitted by the end-device has a FRM Payload size of 51 bytes (left) and 242 bytes (right), respectively.

We next derive the models for obtaining *I_avg_ACK__*_1_ and *I_avg_ACK__*_2_. Figure 7 depicts the current consumption profile of an end-device that performs an acknowledged transmission in two different situations: in Figure 7a), the acknowledgment is received in the second window, while in Figure 7b), the acknowledgment is received in the first window. (Note: in our specific scenario, we observed that for DR0-DR3, all ACKs were received in the second window, while for DR4-DR5 all ACKs were received in the first window.)

When the acknowledgment is received by the end-device in the first window, the number of states involved in acknowledged transmission decreases in comparison with unacknowledged transmission, since the end-device does not need to wait for a second receive window (Table 7). On the other hand, duration of the first receive window (*T_rx_*_1*w*_) and radio off interval (*T_off_*) increase since the acknowledgment needs to be received and subsequently processed. Therefore, *I_avg_ACK__*_1_ can be derived by using the same equations used to compute *I_avg_unACK_* (i.e., Equations (1)–(11)), but considering only the states that exist when the acknowledgment is sent in the first receive window (which is equivalent to setting both *T_w_*_2*w*_ and *T*_2*w*_ to 0 in the equations), and the values in Table 7.

In order to compute *I_avg_ACK__*_2_, we take into account that behavior of the end-device is similar to that in unacknowledged transmission, since states involved in the corresponding transmission operations are the same, and only two differences can be observed: duration of the second window (*T*_2*w*_) and of the subsequent radio off interval (*T_off_*) are both larger than in the unacknowledged transmission. In fact, the end-device needs to stay in the second receive window for the time needed to receive the acknowledgment, and subsequent operations involve processing of the ACK. Therefore, *I_avg_ACK__*_2_ can be obtained by means of the same equations used to compute *I_avg_unACK_* (i.e., Equations (1)–(11)), but using the *T*_2*w*_ and *T_off_* values that correspond to acknowledged transmission when an acknowledgment is sent in the second receive window (Table 8). 

We next extend the model to compute *I_avg_ACK_* for non-zero BER. We assume that bit errors are uncorrelated.

Let *I_k_* denote the average current consumed by the end-device when it performs *k* retransmissions (the last one being successfully acknowledged), since the start of the procedures for the first transmission attempt, until the end of the procedures for the *k*-th retransmission. Let *I_act_* be the average current consumption due to activities related with transmitting a data message (including retransmissions), i.e., all states except the sleep interval. *I_act_* can be computed as:

(19) $$I_{act}=\sum_{k=0}^{MAX\_RETR}\;E\left[I_{k}\right]\cdot p_{k}$$

where *E*[*I_k_*] denotes the expected current consumption of an end-device when it has performed *k* data message retransmissions, *p_k_* indicates the probability that the end-device performs *k* retransmissions of a message, and *MAX_RETR* denotes the maximum number of message retransmissions by an end-device. The latter is recommended as per the LoRaWAN specification to be set to 7.

*E*[*I_k_*] can be obtained by using the next equation:

(20) $$E\left[I_{k}\right]=\frac{I_{OK}^{k}\cdot T_{OK}^{k}+\overset{k}{\underset{i=0}{\sum }}(I_{ACK\_TO}\cdot (T_{ACK\_TO}-T_{rx2w}^{i})+\;\frac{I_{A}^{i}\cdot T_{A}^{i}\cdot p_{A}+I_{B}^{i}\cdot T_{B}^{i}\cdot p_{B}+I_{C}^{i}\cdot T_{C}^{i}\cdot p_{C}}{p_{A}+p_{B}+p_{C}})}{T_{OK}^{k}+\overset{k}{\underset{i=1}{\sum }}(\left(T_{ACK\_TO}-T_{rx2w}^{i}\right)+\frac{T_{A}^{i}\cdot p_{A}+T_{B}^{i}\cdot p_{B}+T_{C}^{i}\cdot p_{C}}{p_{A}+p_{B}+p_{C}})}$$

In the previous equation, $I_{OK}^{i}$ and $T_{OK}^{i}$ denote the average current consumption and average duration of the activities related with an *i*-th acknowledged data transmission attempt which is error-free, and can be computed by using (15) and Table 6, Table 7 and Table 8. On the other hand, *I_ACK_TO_* and *T_ACK_TO_* correspond to the current consumption and the average duration of the *ACK_TIMEOUT* interval, respectively. As per our measurements, *I_ACK_TO_* has the same value as *I_w_*_1*w*_, whereas *ACK_TIMEOUT* is a random variable uniformly distributed between 1 and 3 s. Variables $I_{x}^{i}$, $T_{x}^{i}$ and *p_x_*, where *x* can be equal to *A*, *B* or *C*, correspond respectively to the average current consumption, duration, and probability of unsuccessful acknowledged data message transmission events defined as follows: *A* is the event whereby the data message suffers a collision, or it does not suffer a collision but it suffers at least one bit error, and it is equivalent in terms of current consumption and duration to the active part (i.e., all states minus sleep) in unacknowledged transmission; *B* is the event whereby the data message is successfully received, but the acknowledgment, sent in the first receive window, suffers at least one bit error; and *C* is the event whereby the data message is successfully received, but the acknowledgment sent in the second receive window, suffers at least one bit error (note that errors in downlink messages can be detected by means of the MIC field). Events *B* and *C* are equivalent in terms of current consumption and duration to the active part of successful acknowledged transmission with the acknowledgment in the first and in the second window, respectively. Probabilities *p_A_*, *p_B_* and *p_C_* are determined in Equations (20)–(22). Let *l_Data_* and *l_Ack_* denote the total size of the data and acknowledgment messages, respectively. Probabilities *p_A_*, *p_B_* and *p_C_* can then be obtained as follows:

(21) $$p_{A}=p_{coll}+\left(1-p_{coll}\right)\cdot \left(1-{\left(1-b\right)}^{l_{Data}}\right)$$

(22) $$p_{B}=p_{1win}\cdot \left(1-p_{A}\right)\cdot \left(1-{\left(1-b\right)}^{l_{Ack}}\right)=0.5\cdot \left(1-p_{A}\right)\cdot \left(1-{\left(1-b\right)}^{l_{Ack}}\right)$$

(23) $$p_{C}=p_{2win}\cdot \left(1-p_{A}\right)\cdot \left(1-{\left(1-b\right)}^{l_{Ack}}\right)=0.5\cdot \left(1-p_{A}\right)\cdot \left(1-{\left(1-b\right)}^{l_{Ack}}\right)$$

We next determine *p_k_*. To this end, we first derive the probability that an end-device will send an acknowledged message without performing any retransmissions, *p*_0_. Then, *p*_0_ can be computed as the probability that the data message will not suffer collisions, and neither the data message nor the acknowledgment will suffer errors:

(24) $$p_{0}={\left(1-b\right)}^{l_{Data}}\cdot {\left(1-b\right)}^{l_{Ack}}\cdot \left(1-p_{coll}\right)$$

Based on *p*_0_, *p_k_* can be found as the probability that only both message and acknowledgment transmissions that correspond to the *k*-th message retransmission are successful, as follows:

(25) $$p_{k}={\left(1-p_{0}\right)}^{k}\cdot p_{0}$$

Note that as per Equation (24), when the end-device reaches the maximum number of retransmissions, if the last data message retransmission is not successful, the corresponding current consumption is not added to *I_act_* computation in Equation (19). Nevertheless, impact of this inaccuracy is negligible fo*r MAX_RETR* = 7 and for practical BER values (e.g., up to 10^−3^). Therefore, we opt to favor simplicity in our model.

On the other hand, for non-zero BER, *T_act_* can be calculated as shown next:

(26) $$T_{act}=\overset{MAX\_RETR}{\underset{k=0}{\sum }}\;E\left[T_{act\_k}\right]\cdot p_{k}$$

where *E*[*T_act_k_*] can be determined by using the next equation:

(27) $$E\left[T_{act\_k}\right]=T_{OK}^{k}+\overset{k}{\underset{i=1}{\sum }}\left(\left(T_{ACK\_TO}-T_{rx2w}^{i}\right)+\frac{T_{A}^{i}\cdot p_{A}+T_{B}^{i}\cdot p_{B}+T_{C}^{i}\cdot p_{C}}{p_{A}+p_{B}+p_{C}}\right)$$

Based on Equations (18)–(23), *I_avg_ACK_* can be obtained by considering the active interval and the sleep interval over the notification period, *T_Notif_*, as:

(28) $$I_{avg\_ACK}=\frac{I_{act}\cdot T_{act}+I_{sleep}\cdot \left(T_{notif}-T_{act}\right)}{T_{notif}}$$

The previous equation can be used to calculate the theoretical lifetime (i.e., an upper bound on the actual lifetime) of a battery-operated end-device that performs acknowledged transmissions, denoted *T_lifetime_ACK_*, on the basis of the battery capacity, *C_battery_* (expressed in mA·h), and *I_avg_ACK_*, as shown next:

(29) $$T_{lifetime\_ACK}=\;\frac{C_{battery}}{I_{avg\_ACK}}$$

Finally, we model the energy cost of data delivery in acknowledged transmission, *EC_delivery_ACK_*, which provides the energy consumed by the end-device per each delivered bit of data payload in acknowledged transmission, as shown next:

(30) $$EC_{delivery\_ACK}=\;\frac{I_{avg\_ACK}\cdot V\cdot T_{notif}}{E\left[l_{delivery\_ACK}\right]}$$

where *E*[*l_delivery_unACK_*] indicates the expected amount of data successfully delivered by the end-device per data frame transmitted in acknowledged transmission. 

In acknowledged transmission, the end-device will perform frame transmission retries, upon unsuccessful data frame transmission (either due to collisions or, if no collisions take place, due to bit errors). Therefore, *E*[*l_delivery_ACK_*] is determined as the probability that the data message is successfully delivered in either the first transmission attempt, or one of the retransmissions for the data message:

(31) $$E\left[l_{delivery\_ACK}\right]=\;l_{Pay}\cdot \overset{MAX\_RETR}{\underset{k=0}{\sum }}{\left(\left(1-p_{coll}\right)\cdot \left(1-{\left(1-b\right)}^{l_{Data}}\right)+p_{coll}\right)}^{k}\cdot \left(1-p_{coll}\right)\cdot {\left(1-b\right)}^{l_{Data}}$$

It is worth adding that event *A*, as defined above, captures the reasons that lead to absence of an acknowledgment in response to the data message sent by the end-device (i.e., collisions or bit errors). However, there is a further possible reason for absence of an acknowledgment requested by an uplink data message, which is duty-cycle limitations, since the gateway also needs to comply with regulations on this subject. Lack of an acknowledgment for this reason is likely when there is a sufficiently high amount of downlink traffic (which may comprise acknowledgments to uplink traffic from end-devices and actual downlink data messages to be sent to end-devices). Let *p_NoAckDutyCycle_* denote the probability of absence of an acknowledgment due to duty-cycle limitations. Impact of *p_NoAckDutyCycle_* on the end-device energy performance parameters considered in this paper can be modeled by replacing *p_coll_* by *p_NoAckDutyCycle_* in Equations (21), (24) and (31). Therefore, the evaluation results on the impact of *p_coll_* on performance that are presented in the next section can serve to understand how energy performance of the end-device depends on *p_NoAckDutyCycle_* as well.

## 5. Evaluation

In this section, we use the models derived in Section 4 in order to evaluate LoRaWAN end-device current consumption and lifetime, as well as the energy cost of data delivery. As an additional validation of the evaluation results, we have performed average current consumption measurements of up to 4-min duration (i.e., close to the maximum duration allowed by the power analyzer used), comprising several message transmissions from the end-device, for different configurations in terms of DR, notification period, and acknowledged or unacknowledged transmission. We have found an almost exact match between the average measured current consumption and the one computed by using the analytical models. Note that this is an expected result, since the analytical models have been derived on the basis of measurement results.

The section is divided into three subsections, which focus on each one of the performance parameters mentioned, respectively. Unless explicitly mentioned otherwise, we assume the maximum-sized frame payload for each DR. As it will be shown, this is the most energy-efficient approach per delivered bit, under the assumption that the network comprises a single end-device, and therefore the probability of collision is zero. Nevertheless, the approach of a single-byte payload, which is the least efficient under the same conditions, is also evaluated in the section.

### 5.1. End-Device Current Consumption 

#### 5.1.1. Unacknowledged Transmission

**Figure 8:**
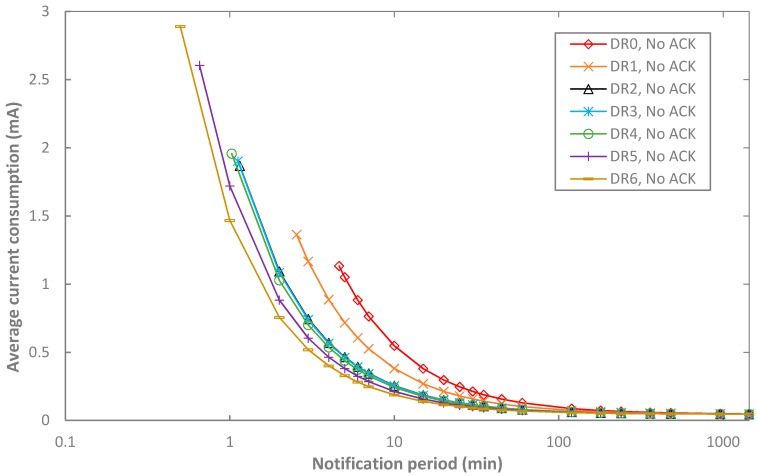
Average current consumption of the end-device in unacknowledged transmission, as a function of *T_Notif_*, and for different DR settings.

We calculate the average current consumption of the end-device in unacknowledged transmission by using the values shown in Table 5 and Table 6 and Equations (1)–(11). Figure 8 illustrates the obtained results, as a function of *T_notif_* and the DR selected. Note that the minimum value for *T_notif_* is given by the 1% duty-cycle limitation that applies for the mandatory, three-channel sub-band, and it depends on the DR.

As expected, the average current consumption decreases as *T_Notif_* increases, since then the sleep intervals have a greater duration, while the duration of the intervals related with communication remains constant. The average current consumption tends asymptotically to the sleep current as *T_Notif_* grows. 

On the other hand, the average current consumption decreases with the DR, since the duration of transmit and receive intervals is inversely proportional to the bit rate of the DR used. For example, the highest relative difference in terms of current consumption between using DR0 and DR5 is a factor of 2.76, for a notification period of 5 min. However, differences due to the DR used decrease with *T_Notif_*. 

Finally, note that, in unacknowledged transmission, end-device current consumption is independent of the BER, as the end-device will perform a single transmission attempt of each data unit, regardless of whether it is correctly received by the network server.

#### 5.1.2. Acknowledged Transmission

**Figure 9:**
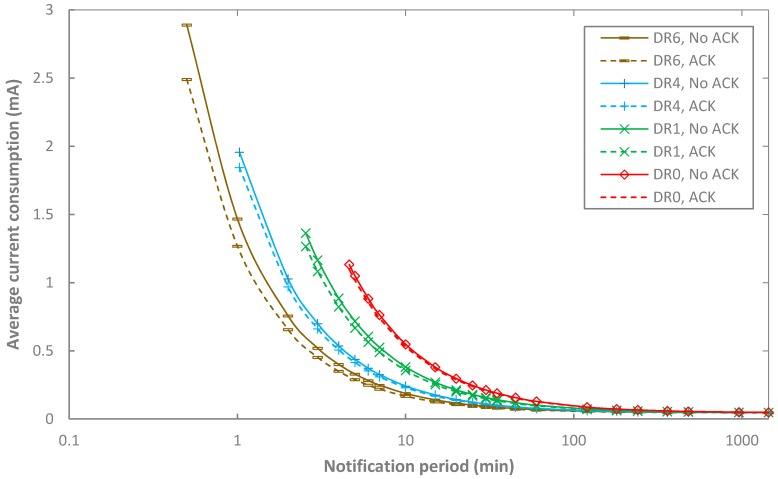
Comparison of the average current consumption of the end-device in acknowledged and unacknowledged transmission, as a function of *T_Notif_*, and for different DR settings, for BER = 0 and *p_coll_* = 0. DR2, DR3 and DR5 are not shown in the figure for the sake of illustration clarity.

We next use the values shown in Table 6, Table 7 and Table 8 and Equations (18)–(28) to compute the average current consumption of the end-device in acknowledged transmission. In order to compare results of unacknowledged and acknowledged transmission, Figure 9 depicts the results, as a function of *T_Notif_*, for various DR settings, and for BER = 0. Since some of the curves of DR2 and DR3 overlap with those of DR4, results for DR2 and DR3 are excluded from Figure 9.

Overall, when BER = 0, the average current consumption of acknowledged transmission follows a tendency with *T_Notif_* that is similar to the unacknowledged transmission one: it decreases with *T_Notif_*, it tends asymptotically to the sleep current, and it decreases with the DR used.

A remarkable result, which is apparently counterintuitive when considering typical behavior of acknowledged link layer protocols other than LoRaWAN, is the fact that acknowledged transmission yields lower current consumption than that of unacknowledged transmission. Note that in acknowledged transmission, the acknowledgment may be received by the end-device in the first or in the second received window. The latter leads to greater average current consumption than in unacknowledged transmission (since the acknowledgment has to be completely received, as opposed to the duration of the second window where no message is received by the end-device). However, the former involves significantly lower current consumption compared to unacknowledged transmission, since then neither a second receive window nor its previous wait time exist. This leads to an overall average current consumption in acknowledged transmission that is lower than the one in unacknowledged transmission. 

The maximum current consumption difference between unacknowledged and acknowledged transmission is ~16%, which is found for DR6 and a notification period of 0.5 min. For other DRs, such maximum difference tends to decrease, down to ~3% for DR0, although such decrease is not monotonical (e.g., the maximum difference for DR4 is lower than that for DR1). Recall that we are considering a frame payload of the highest possible size for each DR, and the maximum frame payload size does not decrease proportionally to the bit rate as the DR decreases. 

It is also interesting to consider the case of acknowledgments being always transmitted in the first receive window (i.e., *p*_1*win*_ = 1 and *p*_2*win*_ = 0). Such conditions lead to the lowest possible LoRaWAN end-device current consumption. In that case, the current consumption in acknowledged transmission decreases by up to 45.1% (for DR5 and a notification period of 1 min), compared to the unacknowledged one. However, such decrease is only significant for low notification periods.

**Figure 10:**
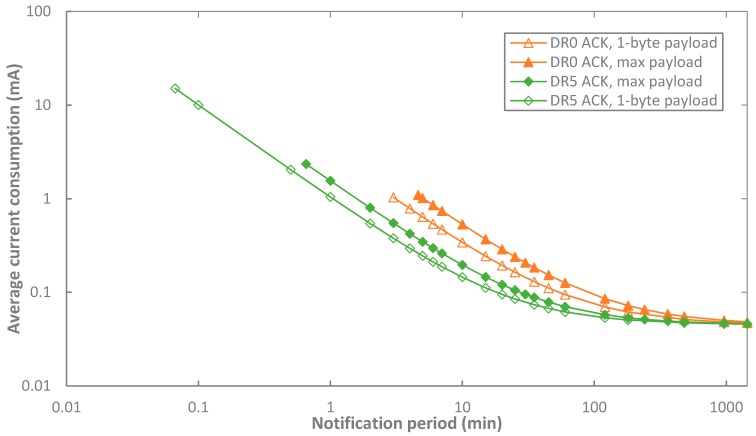
Comparison of the end-device average current consumption with 1-byte and maximum-sized frame payload in acknowledged transmission, as a function of *T_Notif_*, and for different DR settings, for BER = 0 and *p_coll_* = 0. Only DR0 and DR5 are shown in the figure for the sake of illustration clarity.

We also evaluate impact of the frame payload size on current consumption. Figure 10 illustrates the average current consumption of an end-device in acknowledged transmission, for a single-byte and also for a maximum-sized frame payload, which provide the lower and the upper bound on average current consumption for a given DR, respectively. The difference between the lower and upper bounds is significant for a low notification period (e.g., ~49% for a notification period of 1 min, and ~59% for a notification period of 5 min, for DR5 and DR0, respectively). Such difference decreases with *T_Notif_*, since sleep current then becomes dominant, and it also decreases with the DR, since higher bit rates reduce the relevance of active communication states on average current consumption. 

**Figure 11:**
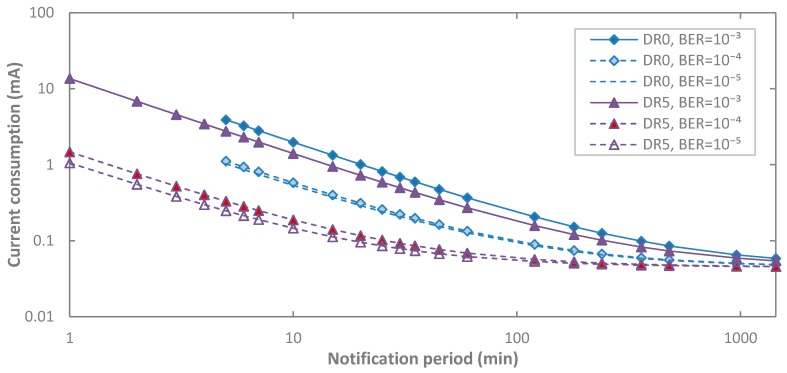
Impact of Bit Error Rate (BER) on the average current consumption of the end-device in acknowledged transmission, as a function of *T_Notif_*, for different DR settings and for *p_coll_* = 0.

We next evaluate end-device average current consumption for non-zero BER in acknowledged transmission, for BER values up to 10^−3^, and for DR0 and DR5. Since for DR5, retransmissions may reduce the DR (see Section 3.3.2), and for the sake of a better comparison, for DR5 we consider the same payload size as that of DR0. As shown in Figure 11, for a given DR, a non-zero BER increases end-device average current consumption (up to one order of magnitude for the range of BER values considered), since the end-device performs retransmissions. This effect increases as *T_Notif_* decreases, since sleep current consumption then becomes less significant. Note that for a given BER, current consumption for DR5 is lower than that when DR0 is used. For DR5, the lower duration of intervals related with transmission and reception reduces current consumption. This phenomenon is emphasized as BER decreases, since then the number of retries decreases as well. 

**Figure 12:**
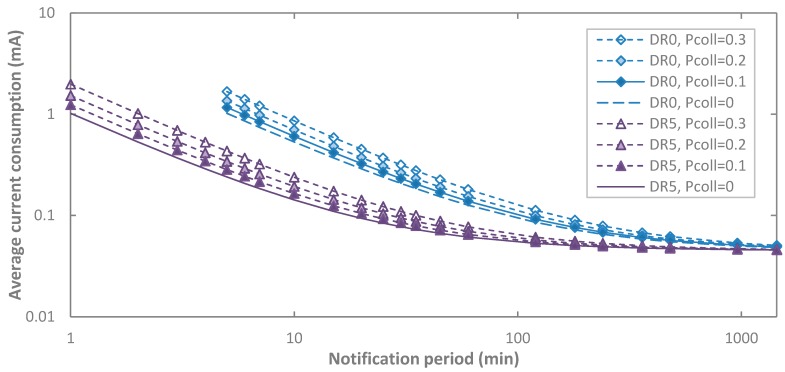
Impact of *p_coll_* on the average current consumption of the end-device in acknowledged transmission, as a function of *T_Notif_*, for different DR settings and for BER = 0.

Finally, we evaluate impact of collisions on the average current consumption of an end-device, for DR0 and DR5, and for *p_coll_* values up to 0.3 (Figure 12). As expected, collisions increase the average current consumption in acknowledged transmission. Increasing *p_coll_* has a slightly greater relative impact on current consumption for DR5 than for DR0. In fact, the contribution to energy consumption of states related with processing and wait times is relatively greater for DR5 (with smaller transmit and receive state duration) than for DR0. Influence of collisions decreases with *T_Notif_*, since sleep current becomes dominant.

### 5.2. End-Device Lifetime 

Leveraging the average current consumption results obtained in the previous section, we next calculate the theoretical lifetime of a battery-operated end-device by using (12) and (29). We assume a battery capacity of 2400 mAh.

**Figure 13:**
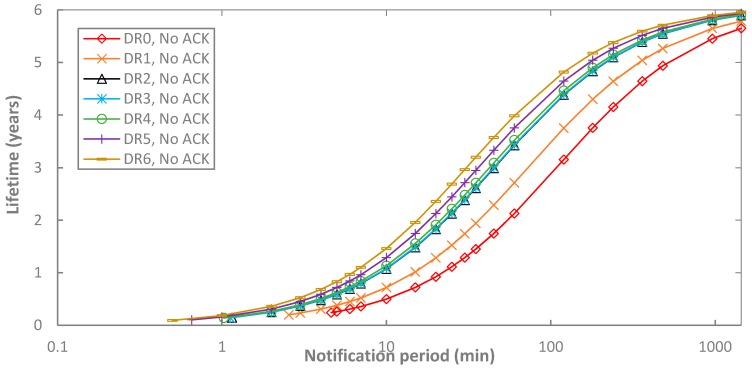
End-device lifetime in unacknowledged transmission, as a function of *T_Notif_*, and for different DR settings.

Figure 13 illustrates the end-device lifetime for BER = 0 as a function of *T_Notif_*, and the DR, in unacknowledged transmission. Logically, the end-device lifetime behavior is the inverse of the current consumption one presented in Section 5.1. A greater end-device lifetime is obtained with higher *T_Notif_* and DR. A maximum value of 5.96 years is obtained with DR6 and *T_Notif_* = 1440 min. In addition, it can be shown that regardless of the DR employed, end-device lifetime tends asymptotically to 6.19 years, which can be achieved if the end-device is always sleeping (i.e., *T_Notif_* = ∞). Note also that lower DRs lead to lower end-device lifetime, and this effect increases as *T_Notif_* decreases. For example, for *T_Notif_* = 5 min, end-device lifetime values fall below one year, ranging from 0.26 to 0.83 years (for DR0 and DR5, respectively). If *T_Notif_* increases, a lifetime of several years is possible. For example, for *T_Notif_* = 60 min and *T_Notif_* = 360 min, multi-year lifetimes between 2.13 and 3.76 years, and between 4.55 and 5.52 years, can be achieved, respectively. 

**Figure 14:**
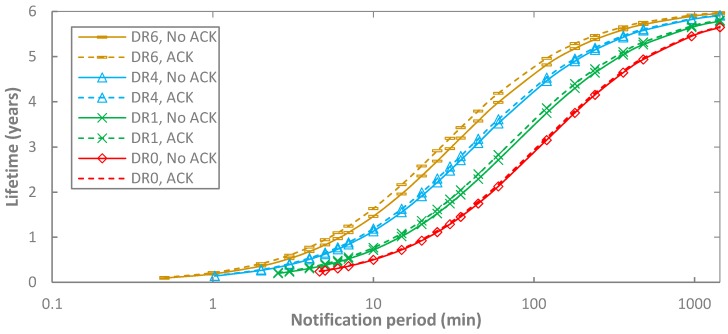
End-device lifetime in acknowledged and unacknowledged transmission, as a function of *T_Notif_*, and for different DR settings, BER = 0 and *p_coll_* = 0. DR2, DR3 and DR5 are not shown in the figure for the sake of illustration clarity.

We next compare end-device lifetime results of unacknowledged and acknowledged transmission (see Figure 14). As explained in Section 5.1.2., acknowledged transmission follows a tendency very similar to the unacknowledged one. The lower current consumption of acknowledged transmission yields higher end-device lifetime. Similarly to Section 5.1.2, the maximum end-device lifetime difference between unacknowledged and acknowledged transmission is ~16%, for DR5 and a notification period of 0.5 min. For other DRs, such maximum difference tends to decrease, down to ~3% for DR0.

**Figure 15:**
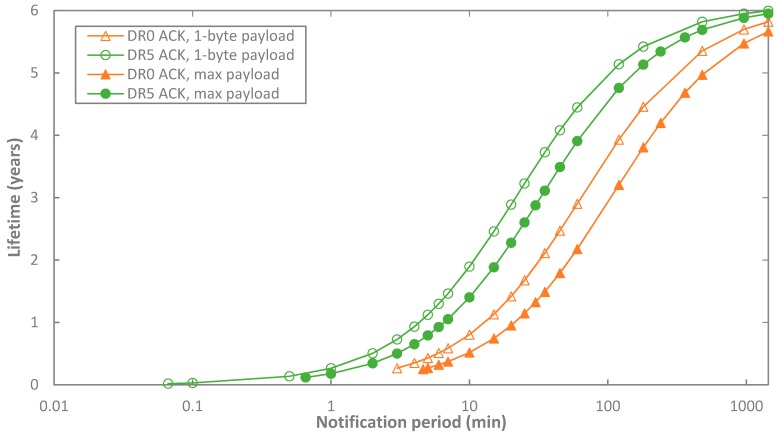
Comparison of the end-device lifetime with 1-byte and maximum-sized payload data message in acknowledged transmission, as a function of *T_Notif_*, for BER = 0, *p_coll_* = 0, and for different DR settings. Only DR0 and DR5 are shown in the figure for the sake of illustration clarity.

On the other hand, Figure 15 shows the impact of the frame payload size on end-device lifetime. In order to obtain the range of possible end-device lifetime values, 1-byte and also maximum-sized payload are considered for data messages in acknowledged transmission. The corresponding end-device lifetimes are depicted in Figure 15, as a function of *T_Notif_*, for different DR settings, and for BER = 0. Only DR0 and DR5 are shown in the figure for the sake of illustration clarity. Impact of the payload size is noticeable only when *T_Notif_* is low. For DR0 and *T_Notif_* = 5 min, end-device lifetime ranges from 0.27 up to 0.41 years (i.e., a ~52% relative difference), for the payload sizes considered. For DR5 and *T_Notif_* = 1 min, end-device lifetime ranges from 0.18 to 0.26 years (i.e., a relative difference of ~49%). However, impact of frame payload size decreases when *T_Notif_* increases because sleep state, and thus its current cconsumption, becomes dominant. For example, for a *T_Notif_* = 60 min and DR0, end-device lifetime values range from 2.17 to 2.90 years (i.e., 33.4% difference), and from 3.90 to 4.44 years (i.e., a 13.78% difference) for DR5. 

**Figure 16:**
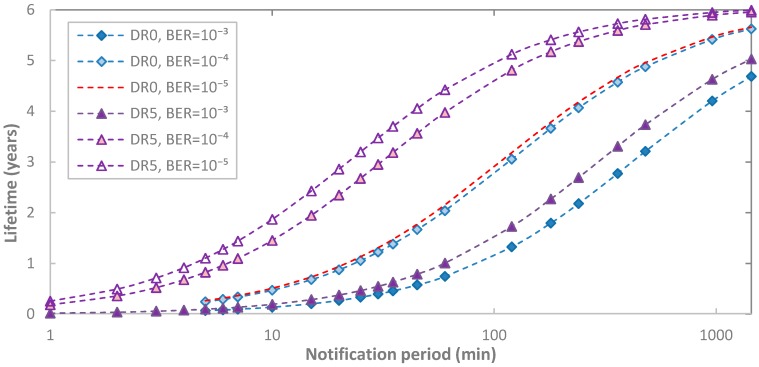
Impact of BER on end-device lifetime in acknowledged transmission, as a function of *T_Notif_*, for different DR settings, and for *p_coll_* = 0.

Next we evaluate impact of BER on end-device lifetime (Figure 16). Similarly to the observations made in Section 5.1.2, end-device lifetime may decrease by up to one order of magnitude for DR5 and for the range of BER values considered. Impact of BER on end-device lifetime is lower for DR0, and it decreases with *T_Notif_*, since end-device lifetime tends asymptotically to the lifetime of an always-sleeping end-device as *T_Notif_* increases. 

**Figure 17:**
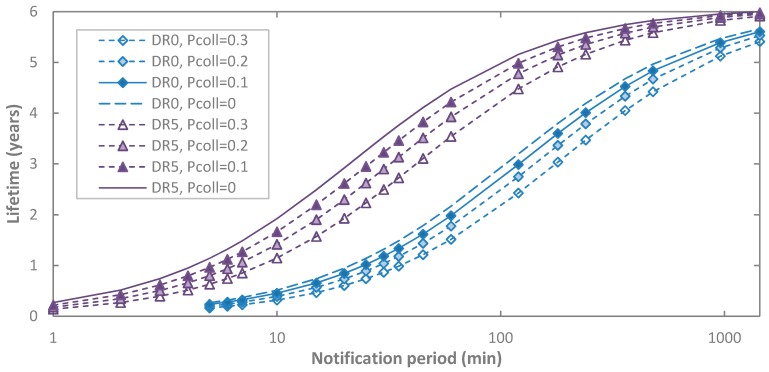
Impact of *p_coll_* on end-device lifetime in acknowledged transmission, as a function of *T_Notif_*, for different DR settings, and for BER = 0.

Finally, we also study the impact of *p_coll_* on end-device lifetime (Figure 17). Collisions may significantly reduce end-device lifetime (e.g., by one third for *T_Notif_* = 30 min and *p_coll_* = 0.3), and have a greater impact on end-device lifetime for DR5, as expected from the average current consumption analysis in Section 5.1.2.

### 5.3. Energy Cost of Data Delivery 

In this subsection, we evaluate the last considered performance parameter, i.e., the energy cost of data delivery for both unacknowledged and acknowledged transmission. We next apply Equations (13), (14), (30) and (31) to determine *E*C*_delivery_*__unACK_ and *E*C*_delivery_*__ACK_, respectively*.* We assume a battery voltage of 3.6 V.

**Figure 18:**
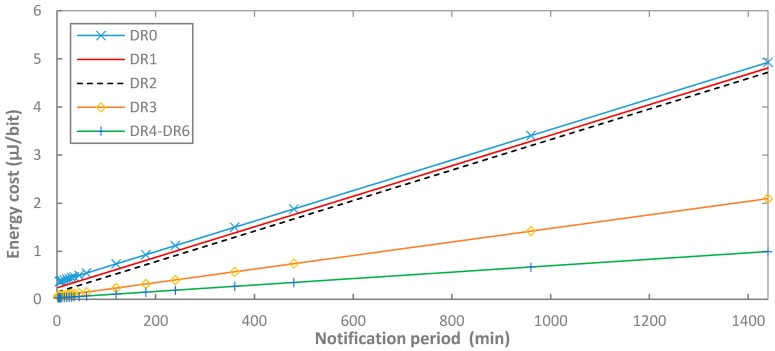
Energy cost of data delivery as a function of *T_Notif_*, for different DR settings, and for BER = 0 and *p_coll_* = 0. Results for DR4, DR5 and DR6 overlap in the figure.

Figure 18 provides the energy cost of data delivery for the unacknowledged approach, as a function of *T_Notif_* and the DR used, for BER = 0. 

For a given DR, and for BER = 0, the relative difference in energy cost of data delivery between acknowledged and unacknowledged transmission is the same as the difference in terms of current consumption analyzed in Section 5.1.2. However, such difference is only significant for low notification periods, and therefore it is not graphically visible in Figure 16, therefore the figure serves for both unacknowledged and acknowledged approaches. 

As shown in Figure 18, for BER = 0, the energy cost of data delivery follows a linear trend as a function of *T_Notif_*. As it has been previously shown in Figure 8 and Figure 9, average current consumption, and therefore energy consumption becomes asymptotically constant as a function of *T_Notif_*. Therefore, as *T_Notif_* increases, the energy consumed by the end-device increases linearly with time, while the number of delivered bits remains constant. Because we are considering the maximum frame payload size allowed by each DR, note that the slope of the curves for DR0-DR2 is the same, since these three DRs allow the same maximum frame payload size (i.e., 51 bytes), the slope for DR3 is lower, since the maximum frame payload size is greater (i.e., 115 bytes), and finally the slope for DR4, DR5 and DR6 is the the lowest, since for these DRs the maximum frame payload size is the largest supported by LoRaWAN (242 bytes). DR0 exhibits slightly greater energy cost of data delivery than DR1, due to the lower bit rate of DR0, which leads to a greater data message and acknowledgment transmit time, as well as receive window duration. The same reasoning applies to the comparison of the energy cost of data delivery of DR1 and DR2. The energy cost of data delivery for DR4 is ~19% and ~40% greater than the one for DR5 and for DR6, respectively, for the lowest notification period, and decreases down to ~1% for both DR5 and DR6 for the highest notification period considered (note that these differences are not visible in Figure 18).

**Figure 19:**
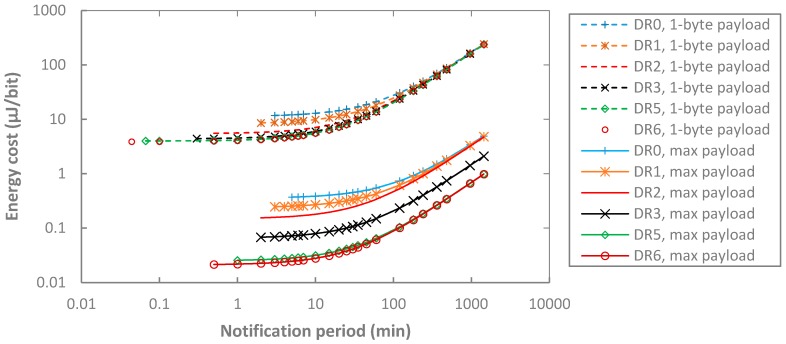
Energy cost of data delivery as a function of *T_Notif_*, for different DR settings, BER = 0, *p_coll_* = 0, and for 1-byte payload and maximum-sized payload that corresponds to each DR.

We next evaluate the upper bound on the energy cost of data delivery by considering a frame payload size of 1 byte. Results are shown in Figure 19, along with the ones obtained for a maximum-sized frame payload. As it can be seen, for a given notification period and DR, the energy cost per delivered payload bit for a 1-byte frame payload is roughly two orders of magnitude greater than the one obtained for a maximum-sized frame payload. 

**Figure 20:**
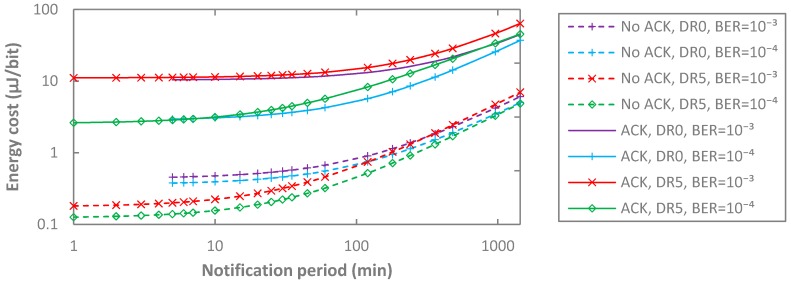
Impact of BER on the energy cost of data delivery, as a function of *T_Notif_*, for both unacknowledged and acknowledged transmission, for *p_coll_* = 0, and for DR0 and DR5.

We then study the impact of non-zero BER on the energy cost of data delivery, assuming a maximum-sized payload for DR0, and the same payload size for DR5, and considering also unacknowledged and acknowledged data message transmission (see Figure 20). Acknowledged transmission leads to a greater energy cost, by at least one order of magnitude, due to the additional energy consumption of retransmissions, in comparison with the unacknowledged approach. On the other hand, BER has a greater impact on DR5 than on DR0, since the former transmits (and may retransmit) data messages at a higher bit rate, leading to lower energy consumption, which makes retransmissions, and their related overhead, energy-expensive.

**Figure 21:**
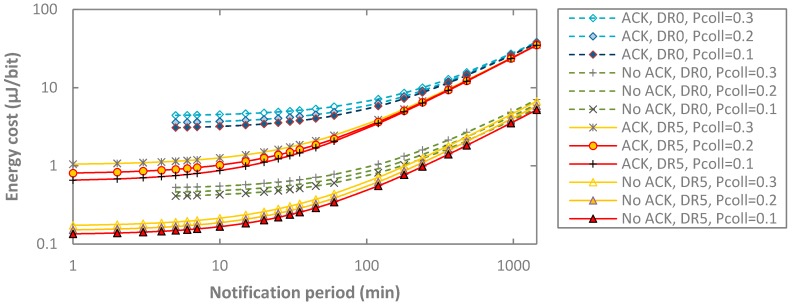
Impact of *p_coll_* on the energy cost of data delivery, as a function of *T_Notif_*, for both unacknowledged and acknowledged transmission, for BER = 0, and for DR0 and DR5.

We next focus on how collisions influence the energy cost of data delivery (see Figure 21). As expected, collisions increase the energy required per delivered payload bit. In acknowledged transmission, this effect is emphasized, since retransmissions are needed. For example, for DR5, the energy cost of data delivery in acknowledged transmission for *T_notif_* = 1 min increases by 60% when *p_coll_* increases from 0.1 to 0.3, while in unacknowledged mode, the energy cost per delivered bit increases by 12.8%.

Finally, we analyze and discuss the impact of payload (and thus, message) size on the energy cost per delivered bit in a dense LoRaWAN network, where collisions may occur. Assuming that end-devices perform acknowledged transmissions, network behavior can be modeled by an Aloha access protocol, as an approximation [7]. Under these conditions, the energy cost per delivered bit can be computed as the result of dividing the energy cost of a message transmission (including its retransmissions) by the number of payload bits carried. The energy consumed in a message transmission (including retransmissions) is roughly proportional to the number of transmission attempts per message. In fact, the amount of energy consumed in each retransmission (which includes the energy consumed over *ACK_TIMEOUT*) is much larger than the energy consumed during the actual transmission state, regardless of the message size, with an accuracy that increases with the DR since transmission time decreases, and with the load offered to the network. On the other hand, in Aloha, the expected number of transmission attempts per message is *e*^2*G*^, where *G* is the total load offered to the network, and thus the expected energy consumed to deliver a message is *a*·*e*^2*G*^, where *a* is the total amount of energy consumed in each transmission attempt. Note that *G* is proportional to the message size, i.e., *l_pay_* + *l_head_*, where *l_head_* denotes the total size of the message headers. Therefore, the energy cost of data delivery under the described conditions can be approximated by (32):

(32) $$EC_{delivery}\approx \frac{a\cdot e^{2G}}{l_{pay}}=\frac{a\cdot e^{k\cdot \left(l_{pay}+l_{head}\right)}}{l_{pay}}=\;\alpha \cdot \frac{e^{k\cdot l_{pay}}}{l_{pay}}$$

where *α* is a constant expressed in Joules, that can be computed by using (33), and *k* is a constant expressed in bit^−1^, which depends on the transmission bit rate and on the total message rate offered to the network.

(33) $$\alpha =a\cdot e^{k\cdot l_{head}}$$

**Figure 22:**
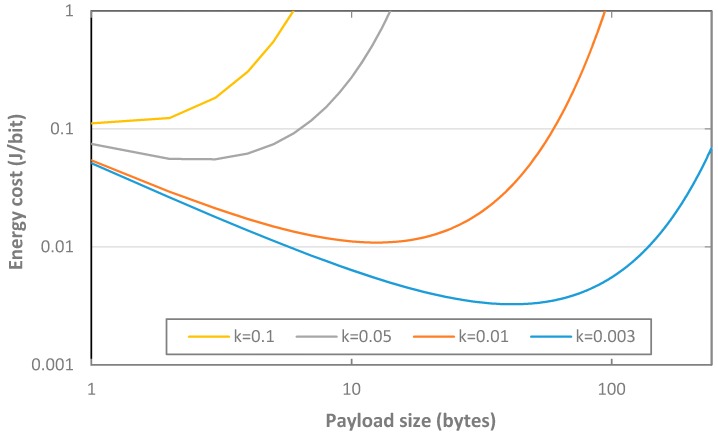
Impact of message payload, *l_pay_*, on the energy cost of data delivery, assuming a dense LoRaWAN network, for, *α* = 0.4 J and DR = 5.

Note that the model presented cannot accurately capture the DR decrease mechanism for retransmissions (see Section 3.3.2), when used, since Aloha assumes all packet transmissions have the same duration. Nevertheless, it allows to qualitatively capture behavior of the energy cost of data delivery as a function of packet size. As shown in Figure 22, impact of payload size on the energy cost per delivered bit depends on the value of *k*. All curves follow a “U” shape with an optimal payload size that minimizes the energy cost per delivered bit. The “U” shape of the curves can be explained, on the one hand, by the fact that very large messages will lead to high energy cost per delivered bit due to a high number of collisions. On the other hand, messages with very small payload will also lead to high energy cost per delivered bit, because while the number of collisions will be relatively low, the energy cost will be relative to a low amount of delivered bits. As *k* increases, impact of collisions becomes dominant and the payload size that minimizes the energy cost per delivered bit decreases. Note that for sufficiently low, or sufficiently high, *k* values, the optimal payload size falls out of the region of valid payload sizes. For example, for a high enough *k* (e.g., *k* = 0.1), the corresponding energy cost per delivered bit curve in Figure 22 is a function that grows steadily with payload size.

## 6. Conclusions

In this paper, we have modeled the energy consumption of a Class A LoRaWAN end-device transmitting data messages periodically, considering impact of unacknowledged and acknowledged transmission, DRs, frame payload size and BER. Performance parameters have been end-device average current consumption and lifetime, and energy cost of data delivery. The models have been developed based on measurements performed on prevalent LoRaWAN hardware.

For BER = 0, acknowledged transmission reduces LoRaWAN end-device average current consumption. This happens because an acknowledgment may be sent in the first receive window, whereas unacknowledged transmission involves two receive windows and a larger average current consumption than a transmission acknowledgment in the first receive window. Note that the quantitative difference between energy consumption in acknowledged and unacknowledged transmission for BER = 0 may be platform-specific. In fact, in contrast with the behavior observed with the platform used in this work, the end-device hardware platform might stay in a low consumption mode (e.g., such as the sleep state) during the interval between the first and the second receive windows. On the other hand, for a non-negligible BER, acknowledged transmission leads to greater current consumption than unacknowledged transmission, due to message retries.

For a notification period of 5 min, DR5 leads to a current consumption lower than that of DR0 by a maximum factor of 2.8, whereas a 1-byte frame payload size reduces current consumption by up to a maximum factor of 1.59. For the same notification period, using DR6 (which is not mandatory as per the LoRaWAN specification), current consumption decreases by a factor of 3.18, compared to using DR0. Non-zero BER up to 10^−3^ may increase current consumption by up to one order of magnitude in acknowledged transmission. Current consumption differences due to the settings considered tend to decrease with the notification period, since sleep current consumption then becomes dominant.

An end-device running on a 2400 mAh battery and sending one message every 5 min can achieve a 1-year lifetime. As the notification period increases, the theoretical end-device lifetime tends asymptotically to roughly 6 years under the conditions considered. However, from our state of the art analysis, we conclude that current LoRaWAN hardware is not as well optimized as that of other low-power technologies. In the latter, sleep current in the order of (or even below) 1 µA is common, which allows multiyear device lifetimes with a button cell battery, of 1 order of magnitude less capacity than the one considered in this study. In contrast, current batteries used for LoRaWAN hardware have a larger size and weight, which in turn has an impact on the physical dimensions and weight of current LoRaWAN devices and limits applicability of current LoRaWAN devices for domains where such dimensions are relevant, such as wearables. 

Finally, the energy cost per delivered bit of maximum-sized frame payload transmission is roughly two orders of magnitude lower than the one obtained for the short payload of 1 byte per frame. An end-device operating as a sensor will thus benefit significantly from accumulating readings and sending them at the highest notification period possible. For non-zero BER up to 10^−3^, acknowledged transmission increases the energy cost of data delivery by up to roughly one and two orders of magnitude, for DR0 and DR5, respectively.

## Figures and Tables

**Table 1:** Current consumption of LoRa/LoRaWAN nodes considered in the literature. When available, transmit power is shown between parentheses next to each corresponding current consumption value in transmit state. Notes: (a) The transmit power consumption setting is not detailed in these papers; we have included datasheet values. (b) A datasheet with power consumption details for this product appears not to be available. (c) The paper does not explicitly indicate the transmit power. We have deduced it based on the current consumption measured (see Table 2). (d) Data are derived from a figure related to power consumption.

Transceiver Included	Device Name	Current Consumption			References
		Sleep	Transmit	Receive	
Semtech SX1272	LoRaWAN Multitech mDot (a) [29]	2 µA 30.9 µA 30.9 µA 40 µA	36 mA	11 mA	[13]
			26–41 mA	12 mA	[14]
			-	-	[15]
			Min.: 26 mA (2 dBm)		
			Max.: 31/41 mA (20 dBm)	-	
Semtech SX1272	NetBlocks XRange [30]	70 µA	109 mA (18.5 dBm)	20 mA	[16]
Microchip RN2483	Microchip RN2483 LoRa Mote (b) [31]	-	38.9 mA (14 dBm)	14.2 mA	[17]
		3.4 mA	47.5–47.9 mA (14.1 dBm)	17.2 mA	[18]
Microchip RN2483	Custom 1 (d)	-	Min.: 23.9 mA (2 dBm)	-	[12]
			Max.: 38.5 mA (14 dBm)		
Microchip RN2483	Custom 2	34 mA	14 dBm: 70 Ma	46 mA	[19]
Semtech SX1276	Custom 3	0.17 mA	Min.: 46 mA (0 dBm)	14 mA	[20]
			Max.: 103 mA (14 dBm)		
Semtech SX1276	LoRaBug [32]	3.7 mA	120.7 mA (20 dBm) (c)	16.6 mA	[21]
HopeRF HM-TRLR-LF/HFS	iLoad (custom)	7.66 μA	133.3 mA (20 dBm) (c)	16.3 mA	[22]

**Table 2:** Main current consumption details on LoRa/LoRaWAN transceivers used by devices in Table 1.

Transceiver	Current Consumption		
	Sleep	Transmit	Receive
Semtech SX1272 [33]	0.1 µA (max. 1 µA)	Min.: 18 mA (7 dBm) Max.: 125 mA (20 dBm)	10.5 or 11.2 mA
Semtech SX1276 [34]	0.2 µA (max. 1 µA)	Min.: 20 mA (7 dBm) Max.: 120 mA (20 dBm)	10.8, 11.5 or 12.0 mA
HopeRF HM-TRLR-LF/HFS [35]	2 µA (min. 1.2 µA, max. 3 µA)	Min.: 35 mA (13 dBm) Max.: 120 mA (20 dBm)	16 mA (min. 15 mA, max. 18 mA)
Microchip RN2483 [36,37]	Up to 100-150 µA	Min.: 17.3 mA (−4.0 dBm) Max.: 38.9 mA (14.1 dBm)	14.2 mA

**Table 3:** Data rates and related configuration for EU863-870 band channels.

Data Rate (DR)	Configuration			Physical Bit Rate (bit/s)
	**Modulation**	**Spreading Factor (SF)**	**Bandwidth**	
0	LoRa	SF12	125 kHz	250
1	LoRa	SF11	125 kHz	440
2	LoRa	SF10	125 kHz	980
3	LoRa	SF9	125 kHz	1760
4	LoRa	SF8	125 kHz	3125
5	LoRa	SF7	125 kHz	5470
6	LoRa	SF7	250 kHz	11,000
7	FSK	50 kbit/s		50,000
8–15	Reserved for Future Use			

**Table 4:** Basic physical layer parameters and their default values.

Parameters		Default Value
Name	Description	
	Downlink data rate, 1st receive window	DR(uplink) minus *RX1DROffset*
	Downlink data rate, 2nd receive window	DR0, SF12
*RX1DROffset*	Data rate offset for the 1st receive window	0
*RECEIVE_DELAY1*	Delay from end of uplink transmission to start of 1st receive window	1 s
*RECEIVE_DELAY2*	Delay from end of uplink transmission to start of 2nd receive window	2 s (must be *RECEIVE_DELAY1* + 1 s)

**Table 5:** States, variables and their values for LoRaWAN unacknowledged transmission.

State Number	Description	Duration		Current Consumption	
		Variable	Value (ms)	Variable	Value (mA)
1	wake up	*T_wu_*	168.2	*I_wu_*	22.1
2	radio preparation	*T_pre_*	83.8	*I_pre_*	13.3
3	Transmission	*T_tx_*	(see Table 6)	*I_tx_*	83.0
4	wait 1st window	*T_w_*_1*w*_	983.3	*I_w_*_1*w*_	27.0
5	1st receive window	*T_rx_*_1*w*_	(see Table 6)	*I*_1*w*_	38.1
6	wait 2nd window	*T_w_*_2*w*_	Equation (4)	*I_w_*_2*w*_	27.1
7	2nd receive window	*T_rx_*_2*w*_	33.0	*I*_2*w*_	35.0
8	radio off	*T_off_*	147.4	*I_off_*	13.2
9	Postprocessing	*T_post_*	268.0	*I_post_*	21.0
10	turn off sequence	*T_seq_*	38.6	*I_seq_*	13.3
11	Sleep	*T_sleep_*	Equation (2)	*I_sleep_*	45 × 10^−3^

**Table 6:** Summary of values for *T_tx_*, *T_rx_*_1*w*_ and *T_rx_*_2*w*_, along with relevant parameter settings. We have assumed an 8-symbol preamble length, a CR of 4/5 (except for the 20-bit physical header, for which a CR of 4/8 is used), and a bandwidth BW = 125 kHz (except for DR6, with a bandwidth BW = 250 kHz, and for DR7, which is based on FSK). *T_tx_* max is obtained by considering the maximum frame payload (FRM Payload) size for each Data Rate (DR), while *T_tx_* min corresponds to the time to transmit a data message that carries no data (e.g., an acknowledgment). In the latter case, the physical frame length is the contribution of the physical header (PHDR) and PHDR_CRC fields, the MAC Header (MHDR), the FHDR and the MIC fields (see Section 3.3.1), leading to a total length of 14.5 bytes plus 8 preamble symbols. Note that the CRC is only present in uplink transmissions (see Section 3.2.2). For DR7, an additional margin should be added to the *T_rx_*_1*w*_ and *T_rx_*_2*w*_ values to account for possible drifts of the oscillator used for the timer that controls a receive window start.

*DR*	*SF*	*T_sym_*	*T_preamble_*	*T*_*rx*1*w*_	*T*_*rx*2*w*_	*DE*	FRM Payload		*T_tx_* Max	*T_tx_* Min
							Max	Min	Uplink	Downlink
		(ms)	(ms)	(ms)	(ms)		(bytes)	(bytes)	(ms)	(ms)
0	12	32.77	401.41	262.14	33.02	1	51	0	2793.5	991.8
1	11	16.38	200.70	131.07	16.64	1	51	0	1560.6	577.5
2	10	8.19	100.35	98.30	8.45	0	51	0	698.4	288.7
3	9	4.10	50.18	49.15	4.35	0	115	0	676.9	144.4
4	8	2.05	25.09	24.58	2.30	0	242	0	707.1	72.2
5	7	1.02	12.54	12.29	1.28	0	242	0	399.6	41.2
6	7	0.51	6.27	6.14	0.64	0	242	0	199.8	20.6
7	-	0.02	0.48	1.28	1.28	-	242	0	42.4	3.2

**Table 7:** States, variables and their values for LoRaWAN acknowledged transmission when the acknowledgment is sent in the first receive window.

State Number	Description	Duration		Current Consumption	
		Variable	Value (ms)	Variable	Value (mA)
1	wake up	*T_wu_*	169.2	*I_wu_*	22.1
2	radio preparation	*T_pre_*	80.4	*I_pre_*	13.7
3	transmission	*T_tx_*	(see Table 6)	*I_tx_*	82.8
4	wait 1st window	*T_w_*_1*w*_	988.4	*I_w_*_1*w*_	27.1
5	1st receive window	*T_rx_*_1*w*_	(*T_tx_ min* in Table 6)	*I*_1*w*_	31.8
8	radio off	*T_off_*	337.8	*I_off_*	13.4
9	postprocessing	*T_post_*	272.5	*I_post_*	20.9
10	turn off sequence	*T_seq_*	37.5	*I_seq_*	13.4
11	sleep	*T_sleep_*	Equation (2)	*I_sleep_*	45 × 10^−3^

**Table 8:** States, variables and their values for LoRaWAN acknowledged transmission when the acknowledgment is sent in the second receive window.

State Number	Description	Duration		Current Consumption	
		Variable	Value (ms)	Variable	Value (mA)
7	2nd receive window	*T_rx_*_2*w*_	(*T_tx_ min* in Table 6)	*I*_2*w*_	38.0
8	radio off	*T_off_*	337.8	*I_off_*	13.4

## References

[1] Raza U., Kulkarni P., Sooriyabandara M. (2017). Low Power Wide Area Networks: An Overview. IEEE Commun. Surv. Tutor..

[2] Minaburo A., Toutain L., Gomez C. (2017). LPWAN Static Context Header Compression (SCHC) and fragmentation for IPv6 and UDP. IETF Internet Draft.

[3] Mikhaylov K., Petajajarvi J., Hanninen T. Analysis of capacity and scalability of the LoRa low power wide area network technology. Proceedings of the 22th European Wireless Conference.

[4] Farrell S. (2017). LPWAN Overview. IETF Internet Draft.

[5] Petajajarvi J., Mikhaylov K., Roivainen A., Hanninen T., Pettissalo M. On the coverage of LPWANs: Range evaluation and channel attenuation model for LoRa technology. Proceedings of the 14th International Conference on ITS Telecommunications (ITST).

[6] Adelantado F., Vilajosana X., Tuset-Peiro P., Martinez B., Melia J. Understanding the limits of lorawan. https://arxiv.org/pdf/1607.08011.

[7] Augustin A., Yi J., Clausen T. (2016). A study of LoRa: Long range & low power networks for the Internet of Things. Sensors.

[8] Nolan K.E., Guibene W., Kelly M.Y. An evaluation of low power wide area network technologies for the Internet of Things. Proceedings of the IEEE International of Wireless Communications and Mobile Computing Conference (IWCMC).

[9] Petajajarvi J., Mikhaylov K., Pettissalo M., Janhunen J., Iinatti J. (2017). Performance of a low-power wide-area network based on LoRa technology: Doppler robustness, scalability, and coverage. Int. J. Distrib. Sens. Netw..

[10] Mikhaylov K., Petajajarvi J., Janhunen J. On LoRaWAN Scalability: Empirical Evaluation of Susceptibility to Inter-Network Interference. https://arxiv.org/pdf/1704.04257.

[11] Haxhibeqiri J., Van den Abeele F., Moerman I., Hoebeke J. (2017). LoRa Scalability: A Simulation Model Based on Interference Measurements. Sensors.

[12] Petajajarvi J., Mikhaylov K., Yasmin R., Hämäläinen M., Iinatti J. (2017). Evaluation of LoRa LPWAN Technology for Indoor Remote Health and Wellbeing Monitoring. Int. J. Wirel. Inf. Netw..

[13] Kim B., Hwang K. (2017). Cooperative Downlink Listening for Low-Power Long-Range Wide-Area Network. Sustainability.

[14] Mahmoud M.S., Mohamad A.A.H. (2016). A Study of Efficient Power Consumption Wireless Communication Techniques/ Modules for Internet of Things (IoT) Applications. Adv. Intern. Things.

[15] Bjelcevic S., Jemson J., Karusala N., Purcell D. LAMBS: Light and Motion Based Safety. https://andyhub.com/wordpress/wp-content/uploads/LAMBSFinalReport.pdf.

[16] Bor M., Roedig U. LoRa Transmission Parameter Selection. Proceedings of the 13th IEEE International Conference on Distributed Computing in Sensor Systems (DCOSS).

[17] Mendívil L.J. (2017). Comparación de Soluciones Basadas en LPWAN e IEEE 802.15.4 Para Aplicaciones de Salud Móvil (“m-Health”). Master’s Thesis.

[18] Neumann P., Montavont J., Noël T. Indoor deployment of low-power wide area networks (LPWAN): A LoRaWAN case study. Proceedings of the IEEE 12th International Conference on Wireless and Mobile Computing, Networking and Communications (WiMob).

[19] Mikhaylov K., Petäjäjärvi J. (2017). Design and implementation of the plug&play enabled flexible modular wireless sensor and actuator network platform. Asian J. Control.

[20] Magno M., Aoudia F.A., Gautier M., Berder O., Benini L. WULoRa: An energy efficient IoT end-node for energy harvesting and heterogeneous communication. Proceedings of the IEEE Design, Automation & Test in Europe Conference & Exhibition.

[21] Dongare A., Hesling C., Bhatia K., Balanuta A., Pereira R.L., Iannucci B., Rowe A. OpenChirp: A Low-Power Wide-Area Networking architecture. Proceedings of the IEEE International Conference on Pervasive Computing and Communications Workshops (PerCom Workshops).

[22] Conus G., Lilis G., Zanjani N.A., Kayal M. An event-driven low power electronics for loads metering and control in smart buildings. Proceedings of the Second International Conference on IEEE Event-Based Control, Communication, and Signal Processing (EBCCSP).

[23] Kim C. AN079-Measuring Power Consumption of CC2530 with Z-Stack. Texas Instruments. http://www.ti.com/lit/an/swra292/swra292.pdf.

[24] Aguilar S., Vidal R., Gomez C. (2017). Opportunistic Sensor Data Collection with Bluetooth Low Energy. Sensors.

[25] Sartori D., Brunelli D. A smart sensor for precision agriculture powered by microbial fuel cells. Proceedings of the IEEE Sensors Applications Symposium (SAS).

[26] Toussaint J., El Rachkidy N., Guitton A. Performance analysis of the on-the-air activation in LoRaWAN. Proceedings of the IEEE 7th Annual Information Technology, Electronics and Mobile Communication Conference (IEMCON).

[27] Sornin N., Luis M., Eirich T., Kramp T., Hersent O. (2016). LoRaWAN Specification.

[28] LoRaWAN™ Certified Products. https://www.lora-alliance.org/certified-products.

[29] LoRaWAN Multitech mDot. http://www.multitech.com/documents/publications/data-sheets/86002171.pdf.

[30] NetBlocks XRange. https://www.netblocks.eu/xrange-sx1272-lora-datasheet/.

[31] Microchip RN2483 LoRa Mote. http://www.microchip.com/DevelopmentTools/ProductDetails.aspx?PartNO=dm164138.

[32] LoRaBug. https://github.com/OpenChirp/LoRaBug.

[33] Semtech SX1272. http://www.semtech.com/images/datasheet/sx1272.pdf.

[34] Semtech SX1276. http://www.semtech.com/images/datasheet/sx1276_77_78_79.pdf.

[35] HopeRF HM-TRLR-LF/HFS Series 100 mW. http://www.hoperf.com/upload/rf/HM-TRLR-S_Series_english_.pdf.

[36] Microchip RN2483. http://ww1.microchip.com/downloads/en/DeviceDoc/50002346A.pdf.

[37] Errata Microchip RN2483. http://ww1.microchip.com/downloads/en/DeviceDoc/80000689A.pdf.

[38] Semtech Corporation (2013). SX1272/3/6/7/8: LoRa Modem. Designer’s Guide. AN1200.13.

[39] LoRa Alliance Technical committee (2017). LoRaWAN™ 1.0.2 Regional Parameters.

[40] Kerlink LoRa IoT Station. http://www.kerlink.fr/images/Kerlink/fiches_produit/LoRa-IoT-Station.pdf.

[41] Seller O., Sornin N. (2014). Low Power Long Range Transmitter. U.S. Patent.

